# ALFA-PRF: a novel approach to detect murine perforin release from CTLs into the immune synapse

**DOI:** 10.3389/fimmu.2022.931820

**Published:** 2022-12-22

**Authors:** Jesse A. Rudd-Schmidt, Romain F. Laine, Tahereh Noori, Amelia J. Brennan, Ilia Voskoboinik

**Affiliations:** ^1^ Killer Cell Biology Laboratory, Peter MacCallum Cancer Centre, Melbourne, VIC, Australia; ^2^ Medical Research Council (MRC)-Laboratory for Molecular Cell Biology, University College London, London, United Kingdom; ^3^ The Francis Crick Institute, London, United Kingdom; ^4^ MicrographiaBio, Translation & Innovation Hub, London, United Kingdom; ^5^ Sir Peter MacCallum Department of Oncology, University of Melbourne, Parkville, VIC, Australia

**Keywords:** cytotoxic lymphocyte, granzyme, immunological synapse, cytotoxic granules, trafficking, microscopy

## Abstract

When killing through the granule exocytosis pathway, cytotoxic lymphocytes release key effector molecules into the immune synapse, perforin and granzymes, to initiate target cell killing. The pore-forming perforin is essential for the function of cytotoxic lymphocytes, as its pores disrupt the target cell membrane and allow diffusion of pro-apoptotic serine proteases, granzyme, into the target cell, where they initiate various cell death cascades. Unlike human perforin, the detection of its murine counterpart in a live cell system has been problematic due its relatively low expression level and the lack of sensitive antibodies. The lack of a suitable methodology to visualise murine perforin secretion into the synapse hinders the study of the cytotoxic lymphocyte secretory machinery in murine models of human disease. Here, we describe a novel recombinant technology, whereby a short ALFA-tag sequence has been fused with the amino-terminus of a mature murine perforin, and this allowed its detection by the highly specific FluoTag^®^-X2 anti-ALFA nanobodies using both Total Internal Reflection Fluorescence (TIRF) microscopy of an artificial synapse, and confocal microscopy of the physiological immune synapse with a target cell. This methodology can have broad application in the field of cytotoxic lymphocyte biology and for the many models of human disease.

## 1 Introduction

Cytotoxic lymphocytes (cytotoxic T lymphocytes and natural killer cells) are responsible for the immune surveillance of intracellular infections and transformed cancerous cells. The lymphocyte recognises a target cell, forms an immune synapse and releases toxic cargo of effector proteins, the pore forming protein, perforin, and pro-apoptotic serine proteases, granzymes (1). Perforin forms transmembrane pores specifically on the target cell (2, 3), and this allows diffusion of granzymes into the target cell cytosol, where they initiate various apoptotic cascades.

The most conventional strategies for studying immune interactions between cytotoxic lymphocytes and their targets are bulk assays - various cytotoxicity tests and a degranulation (CD107a externalisation) assay - which are limited to averaged values over thousands of effector-target interactions and immunological synapses. Whilst these assays certainly have their place, spatio-temporal information, which can elucidate details of individual synapses that otherwise remain hidden, is often required to better understand biological mechanisms. Thus, single cell microscopy of synapse formation has created key findings in multiple fields of T cell biology research (4–9). The detection of granule components released from individual cells has also been achieved with high resolution, mostly in fixed samples (10, 11) or once granule components have been captured on a stimulatory surface and the cytotoxic lymphocytes removed (12, 13), and only recently have they been detected using live human cells (14).

Time-lapse microscopy has identified the exact moment of perforin and/or granzyme delivery to the target cell and the initiation of apoptotic cascades (15). This allowed for temporal measurements of key events that determine the formation of a functional immune synapse, such as target cell recognition by a lymphocyte as determined by calcium flux, perforin-mediated target cell permeabilization, granzyme-mediated apoptosis, lymphocyte detachment from the target, serial killing and others (2). Importantly, these *in vitro* time-lapse microscopy experiments have relied on an indirect measurement of perforin pore formation (influx of Propidium Iodide) to identify murine perforin release. Other single cell microscopy studies have also demonstrated the ability of cytotoxic lymphocytes to perform serial killing *in vivo* (16), and shown a role for additive cytotoxicity of CTLs when targeting non haematological solid tumours (17).

Of course, an ideal system would allow the detection of unmodified endogenous proteins, but in live cell imaging this is technically challenging. For human T (14) and NK cells (12), fluorescently labelled anti-perforin antibody (clone δG9) was used to detect secreted endogenous perforin within SupraMolecular Attack Particles (SMAPs). Detection of human perforin within target cells after NK cell attack has also been demonstrated, however the authors concede this was only seen in ‘a few’ cells (18). Another study utilised the combination of two anti-human perforin antibody clones (CE2.10, δG9) as a coverslip bound capture system to detect perforin once it had been secreted from NK cells (13). However, for murine perforin, these same systems cannot be applied successfully due to the low abundance of perforin in T cells (19) and the lack of sufficiently sensitive antibodies. Indeed, the clearest imaging of murine perforin to date has required Bouin’s fixation and antibody staining for 16 hours at 4°C (19). Although the use of human cells is of course the most relevant to understanding human disease, many experimental systems almost exclusively rely on murine models.

It has been well documented that the unstructured carboxy-terminus of perforin is important for protecting the cell from perforin cytotoxicity (20, 21) and, at the same time, it is proteolytically cleaved inside the secretory vesicles (21, 22). Therefore, any “traditional” carboxy-terminal fusion of perforin with a reporter peptide is likely to be cleaved inside the secretory vesicles prior to its release into the synaptic cleft.

In order to bypass the problematic carboxy-terminal processing of perforin, we have developed a novel strategy, where we engineered a stable, short and compact (α-helical) ALFA-tag peptide (SRLEEELRRRLT) (23) at its amino-terminus, immediately after the signalling peptide (24). We found that, when secreted outside CD8 T cells, ALFA-PRF fusion protein is recognised by highly specific fluorescently labelled nanobodies, and can be detected within artificial and physiological immune synapses formed, respectively, on anti-CD3/CD28 coated glass coverslips (using TIRF microscopy) and antigen-presenting target cells (using spinning disk confocal microscopy). This approach opens an exciting array of possibilities for investigating fundamental and applied biology of cytotoxic lymphocytes.

## 2 Materials and equipment

### 2.1 Generation of DNA constructs

a. Synthesis of ALFA-PRF-WT and ALFA-PRF-TMH (Integrated DNA Technologies)
b. MSCV-IRES-TagBFP plasmid
*This is an MSCV-IRES-GFP template (Addgene, Plasmid #20672), where GFP was replaced with TagBFP*
c. Lifeact-mScarlet fusion possessing 5’ EcoR1 and 3’ Age1 restriction enzyme cut sites (Integrated DNA Technologies)

### 2.2 Transfection of Hek cells

a. Maxi Prep DNA stocks of both packaging vectors and DNA constructs at around 5µg/µl
b. CaCl_2_ 1M
c. HEBS Buffer (HEPES buffered saline)

### 2.3 Transduction of primary Murine T cells

a. Non T.C. treated 6 well plates
b. Retronectin (Takara Bio, Shiga, Japan)
c. PBS -/- (no Ca2+/Mg2+)
d. 0.45µM Filters
e. RPMI-1640 media (Gibco, Massachusetts, USA)
f. Heat Inactivated Foetal calf serum (hi-FCS)
g. GlutaMAX™ (Gibco, Massachusetts, USA)
h. Penicillin/Streptomycin
i. Sodium Pyruvate
j. Non-Essential Amino Acids
k. 2-mercaptoethanol
l. Recombinant Human Il-2
m. SIINFEKL peptide

### 2.4 Flow cytometry sorting of transduced T cells

a. Flow cytometry sorting machine equipped with both Blue (405nm) and Red (561nm) lasers

### 2.5 Live cell TIRF imaging of T cells upon CD3/CD28 activation in presence of FluoTag^®^-X2 anti-ALFA nanobodies

a. Ibidi µ-Slide 18 well 1.5H glass bottom chamber wells (Ibidi, Martinsried, Germany)
b. Purified Hamster Anti-Mouse CD3ε (Clone 145-2C11) (BD Biosciences, New Jersey, USA)
c. Purified Hamster Anti-Mouse CD28 (Clone 37.51) (BD Biosciences, New Jersey, USA)
d. DMEM media (Gibco, Massachusetts, USA)
e. Heat Inactivated Foetal calf serum (hi-FCS)
f. GlutaMAX™ (Gibco, Massachusetts, USA)
g. FluoTag^®^-X2 anti-ALFA Atto 488 OR Alexa-647 nanobodies (NanoTag Biotechnologies GmbH, Göttingen, Germany)
h. Zeiss Elyra microscope (Or any microscope with dual/tri colour live cell TIRF capabilities)

### 2.6 Live cell spinning disk confocal imaging of T cell-target cell interactions in the presence of FluoTag^®^-X2 anti-ALFA nanobodies

a. Ibidi µ-Slide 18 well 1.5H glass bottom chamber wells (Ibidi, Martinsried, Germany)
b. SIINFEKL peptide
c. Lifeact-eGFP EL4 target cells
d. DMEM media (Gibco, Massachusetts, USA)
e. Heat Inactivated Foetal calf serum (hi-FCS)
f. GlutaMAX™ (Gibco, Massachusetts, USA)
g. FluoTag^®^-X2 anti-ALFA Alexa-647 nanobodies (NanoTag Biotechnologies GmbH, Göttingen, Germany)
h. Nikon SoRA spinning disk microscope (Or any microscope with fast three colour live cell z-stack imaging capabilities)

### 2.7 Image processing and analysis

a. Zen Black and Zen Blue software
*Or the software interface of whichever microscope system is used to capture the images*
b. FIJI/ImageJ software
c. Imaris software
d. Microsoft Excel software
e. Graph pad Prism software

## 3 Methods

### 3.1 Generation of DNA constructs

a. Clone ALFA-PRF-WT cDNA into an MSCV-IRES-TagBFP plasmid between EcoR1 and Xho1 restriction digest sites.
b. Clone ALFA-PRF-TMH cDNA into an MSCV-IRES-TagBFP plasmid between EcoR1 and Xho1 restriction digest sites.
c. Clone Lifeact-mScarlet fusion sequence into MSCV-IRES-GFP (Addgene, Plasmid #20672) between EcoR1 and Age1 (hence removing the fluorescent reporter of the vector)

*Avoid the use of Sal1 enzyme for Lifeact-mScarlet cloning as mScarlet possesses an internal Sal1 cut-site.*

### 3.2 Transfection of HEK293T cells

Utilise standard calcium phosphate transfection (25) of HEK293T cells to produce viral supernatants of the following constructs: MSCV-IRES-TagBFP, MSCV-ALFA-PRF-WT-IRES-TagBFP, MSCV-Lifeact-mScarlet.

### 3.3 Transduction of primary murine CD8+ T cells

a. Utilise standard CTL transduction protocol (26) to transduce freshly isolated OTI splenocytes. To create double transduced cells, make 1:1 combinations of viral supernatents (either Lifeact-mScarlet + Empty TagBFP, or Lifeact-mScarlet + ALFA-PRF-WT-TagBFP) before addition of viral supernatant to the retronectin plate.
b. Stimulate T cells with 10nM SIINFEKL peptide and 100U/ml Il-2 in in T cell media (RPMI-1640 supplemented with 10% hi-FCS, 2 mM GlutaMAX™, 1mM sodium pyruvate, 100 µM non-essential amino acids, 50 µM 2-mercaptoethanol and penicillin/streptomycin)for 3 days at 37°C and 5% CO_2_.
c. After 3 days, wash the cells 3x at 500xg (4 minutes) and put back into culture at 500,000 cells/ml in T cell media containing 100U/ml Il-2.

### 3.4 Flow cytometry sorting of transduced T cells

Both the Empty TagBFP and ALFA-PRF-WT-TagBFP expressing cells are cell-sorted for equal fluorescence intensity of the TagBFP reporter and Lifeact-mScarlet.

a. Collect TagBFP/Lifeact-mScarlet double positive cells (405/561nm excitation laser wavelength)
b. Set sort gates to collect double positive cells with approximately equal mScarlet and TagBFP mean fluorescence intensity (MFI) from both empty vector and ALFA-PRF-WT expressing cells.
*We have observed an unusual phenomenon whilst using the combination of very bright Lifeact-mScarlet and the TagBFP constructs - a severe (and unusual) bleed through from mScarlet to BFP channels. It is critical to run single colour controls of each fluorophore to allow for appropriate compensation. Please see* **Supplementary Figure 1** *for details. In the case that only perforin and actin need to be visualised, we suggest considering alternative fluorophore combinations of Lifeact-GFP and FluoTag^®^-X2 anti-ALFA Alexa 647.*
c. Wash sorted cells twice in complete media (500xg, 4 minutes)
d. Resuspend at 500,000 cells/ml in T cell media supplemented with 100U/ml Il-2, and continue culturing at 37°C and 5% CO_2_

### 3.5 Live cell TIRF imaging of T cells upon CD3/CD28 activation in presence of FluoTag^®^-X2 anti-ALFA nanobodies

a. Preparation of the anti-CD3/CD28 coated glass coverslip bottom chamber

i. On the day before the experiment, coat Ibidi µ-Slide 18 well 1.5H glass bottom chamber wells (Ibidi, Martinsried, Germany) with 100µl of anti-CD3 (10µg/ml)/CD28 (5µg/ml) in PBS -/-. Leave at 4 degrees overnight.
ii. On the day of the experiment wash the anti-CD3/CD28 coated wells twice with 200µl of PBS -/- (no Ca^2+^/Mg^2+^)
iii. Transfer the Imaging chamber to the slide holder on the Zeiss Elyra microscope
iv. Turn on Tokai Hit microscope heating stage and ensure C0_2_ is supplied
v. Select a 100x lens (alpha Plan-Apochromat 100x/1.46 Oil DIC M27 Elyra) and apply lens oil (optimised for use at 37°C)
vi. Move lens/oil into contact with the bottom of the glass coverslip bottom chamber slide
vii. Allow microscopy chamber to heat equilibrate for at least 1 hour
viii. Transport a heat block to a bench located close to the microscope and set to 37°C

b. Preparation of the Microscope for TIRF imaging

i. Set up the microscope to image in TIRF mode using 3 separate colour channels (488nm, 561nm and 642nm laser lines)
ii. Set laser power as appropriate, in our system these settings were as follows:
1. Track 1 (488nm laser): 0.3%
2. Track 2 (561nm laser): 0.1%
3. Track 3 (642nm laser): 0.4%
iii. Set tile scan to 2x2 (25% overlap, no online stitching applied)
iv. Set frame average 2
v. Set zoom to 1
vi. It is difficult to describe optimal TIRF angles that will work across all systems, however the settings used in our experiments were as follows:
1. Track 1 (488nm laser): TIRF mirror angle = 62.49 Collimator = 2019
2. Track 2 (561nm laser): TIRF mirror angle = 61.97 Collimator = 2022
3. Track 3 (642nm laser): TIRF mirror angle = 61.97 Collimator = 2079
vii. We advise the user to run a trial experiment at the start of the day, to optimise the detection of secreted proteins by altering the TIRF mirror angle and TIRF collimators.

c. Preparation of the nanobody/antibody solution

i. Dilute to 50nM (1:50 stock dilution) FluoTag^®^-X2 anti-ALFA Atto 488 nanobodies (NanoTag Biotechnologies GmbH, Göttingen, Germany) in complete Gibco DMEM (10% FCS, 2mM GlutaMAX™)
ii. Keep an appropriate amount of these master stocks in the dark on ice, so they can be used throughout the imaging session across multiple samples
*When using Ibidi µ-Slide 18 well 1.5H glass bottom chamber wells this technique uses 100µl of antibody mix per sample.*

d. Preparation of the cells for imaging

i. Count T cells from each group
ii. Transfer 250,000 cells to a 1.5ml epindorf tube
iii. Centrifuge cells (500xg, 4 min) and resuspend in 150µl of the nanobody/antibody mix
iv. Incubate cells at 37°C for 10 minutes (ideally on a heat block located in close proximity to the microscope, to reduce thermal drift of the microscope stage upon addition of the cells to the imaging chamber)

e. Imaging release of perforin from T cells *via* TIRF

i. Remove the PBS from the chamber above the lens and immediately replace with the cells which have been kept at 37°C
ii. Set timer for 3 minutes
iii. In these 3 minutes, whilst the cells sediment on the bottom of the chamber, close the lid to the microscope and locate the cells using the bright field light
iv. Turn on the 561nm laser line and focus on the cells
*To obtain an accurate focus at the plain of the coverslip in this early stage of imaging, focus on the small punctate signals in the lifeact-mScarlet signal corresponding to the scanning microvilli of the T cells reaching down to interact with the coverslip (See* **Supplementary Figure 2** *)*
v. After 3 minutes has elapsed and the cells are in focus, take the first tile image by selecting ‘Start Experiment’. This is t=0
vi. Set timer for 45 minutes
vii. Image at regular timepoints for the remaining 45 minutes, manually focusing the lifeact-mScarlet signal before each image is acquired (For this study, images were taken every 5 minutes)

### 3.6 Live cell spinning disk confocal imaging of T cell-target cell interactions in the presence of FluoTag^®^-X2 anti-ALFA nanobodies

a. Preparation of the Lifeact-GFP EL4 target cells

i. On the day of the experiment label approximately 2 million Lifeact-GFP expressing EL4 cells with 1μM SIINFEKL for 1 hour at 37°C.
ii. After 1 hour, wash cells 3 times with complete media
iii. Resuspend cells at 1 million/ml and place in one well of a 12 well plate. This becomes the stock of SIINFEKL labelled lifeact-GFP expressing EL4 target cells which can be used for the remainder of the imaging session. *Because the target cells are adhered using serum free media, it is necessary to adhere the target cells separately before each well/experiment is imaged.*

b. Preparation of the Microscope for time lapse Z stack imaging.

i. Turn on microscope heating stage to 37°C and ensure C0_2_ is supplied
ii. Allow microscopy chamber to heat equilibrate for at least 2 hours
iii. Select a 60x oil lens (Plan Apo λ 60x Oil) and apply lens oil
iv. Transfer an Ibidi µ-Slide 18 well 1.5H glass bottom chamber well imaging chamber to the slide holder
v. Move lens/oil into contact with the bottom of the glass coverslip bottom chamber slide
vi. Set up the microscope to image in spinning disk confocal mode (single sona mode) using 3 separate colour channels (488nm, 561nm and 642nm laser lines).
vii. Set laser power as appropriate, in our system these settings were as follows:
1. Track 1 (488nm laser): 5%
2. Track 2 (561nm laser): 15%
3. Track 3 (638nm laser): 30%
viii. Set exposure times as appropriate, in our system these settings were as follows:
1. Track 1 (488nm laser channel): 100ms
2. Track 2 (561nm laser channel): 100ms
3. Track 3 (638nm laser channel): 100ms
ix. Set binning to 2x2

c. Preparation of the nanobody/antibody solution

i. Dilute to 50nM (1:50 stock dilution) FluoTag^®^-X2 anti-ALFA Atto 488 nanobodies (NanoTag Biotechnologies GmbH, Göttingen, Germany) in complete Gibco DMEM (10% FCS, 2mM GlutaMAX™)
ii. Keep an appropriate amount of these master stocks in the dark on ice, so they can be used throughout the imaging session across multiple samples
*When using Ibidi µ-Slide 18 well 1.5H glass bottom chamber wells this technique uses 100µl of antibody mix per sample.*

d. Preparation of the cells for imaging

i. Take 200µl (200,000 cells) of the SIINFEKL pulsed Lifeact GFP EL4 target cells and centrifuge in a 1.5ml epi tube
ii. Resuspend in 100μl of Serum Free media
iii. Add this 100μl to one well of the 18 well imaging chamber
iv. Allow to incubate on the microscope at 37°C for 20 minutes
v. In this time, transfer 25,000 Cherry Tubulin expressing T cells to a 1.5ml epindort tube
vi. Centrifuge cells (500xg, 4 min) and resuspend in 100µl of the nanobody/antibody mix

e. Imaging release of perforin from T cells *via* Spinning Disk Confocal

i. Remove the serum free media from the Lifeact GFP EL4 cells (which will have now adhered to the bottom of the chamber) and immediately replace with the Cherry tubulin expressing T cells in presence of Fluo-Tag X2 Anti-ALFA 647
ii. Set the microscope to perform a 13.5μm Z-stack with 300nm step size, centred on the middle of the EL4 target cells
iii. Choose ‘no delay’ time interval setting, which depending on the exact imaging settings should image an entire z-stack at least once every minute
iv. Run experiment for 1-1.5 hours, then repeat in a new well with freshly adhered target cells.

### 3.7 Image processing and analysis

a. Within Zen Black program, open the tiled images and stitch them together using ‘Stitch’ function
b. Open these Stitched images within Zen Blue software and export the images in bulk as OME TIFFs
c. Open the OME TIFF images from the chosen experimental timepoint within FIJI/ImageJ software
d. Separate the individual colour channels of the images

#### 3.7.1 Image-Stacks–’Stack to Images’

e. Using the Lifact-mScarlet channel, manually draw cell borders across the entire field of view, and save these regions for each sample using the Region Of Interest (ROI) manager
*At this point, blind the samples before any cell regions are drawn (to avoid potential bias). Adjust the brightness/contrast display of the actin image as you draw the regions to allow for identification of both bright and dull cells*
f. To avoid measuring perforin signal from cells that are partially obscured by the edge of the field of view, note for later exclusion any of these border regions
*Once any regions located on the edge of the field of view have been noted for exclusion in an unbiased way, the samples can be unblinded*
g. Open the ALFA-PRF (Atto 488) channel of ‘Empty Vector’ control image and determine the highest pixel value present within the synapse regions of this group. To do this, go to ‘set measurements’ and select ‘Min/max grey value’ and then measure the regions. From the results, choose the highest grey value within the regions as the threshold level to subsequently apply to the ‘ALFA-PRF’ images
*When creating display images, this threshold value can also be used as the lower bound for brightness/contrast settings, to avoid the display of dull background signal from unbound FluoTag^®^-X2 anti-ALFA nanobodies (which is always present but much duller than the signal of bound Fluo-Tag^®^ X2 Anti ALFA nanobodies).*
h. Before applying the threshold to the samples of interest, it is necessary to apply the following global setting on FIJI/ImageJ:
*If ‘black background’ is left selected, the later measurements will not correctly ‘limit to threshold’, giving false area values for the perforin signal.*

#### 3.7.2 Process – Binary – Options – Uncheck ‘black background’

i. Now the threshold value can be applied to create an 8-bit image of the ALFA-PRF channels for the other samples of interest. First create a duplication of the image, and then apply the threshold so that the only signal left in these images is that which is brighter than the brightest signal in the empty vector control:
*Proceed immediately from this threshold step to the area measurement, without saving and re-opening the image. Depending on the local settings of the FIJI/Image J program, measuring from the previously saved image can affect the ‘limit to threshold’ function.*

#### 3.7.3 Adjust – Threshold – Set – Apply

j. Transfer the previously saved cell border regions to the ALFA-tag perforin channel using the ROI manager
k. Specify measurement parameters:

#### 3.7.4 Set measurements – check both ‘Analyse Area’ and ‘limited to threshold’

l. Measure these values for each of the samples and collect the data in a Microsoft excel spreadsheet, clearing the FIJI/Image J measurement results table after each sample.
m. Finally, within Microsoft excel, exclude any regions which you have previously marked for exclusion (Step 6g), and transfer the remaining values into a Graphpad Prism file to be plotted as a Column graph
n. Final data will be a measure of perforin area per cell
*If a final value of Perforin signal as a percentage of cell area is preferred, simply perform two measurements, with and without ‘Limited to threshold’ applied. Then divide the ‘limited to threshold’ value by the not limited to threshold value and multiply by 100.*
o. (Optional) If AVI movies are required to display the perforin release over time, the following workflow can be followed:
i. Separate each experiment group’s OME TIFF’s into individual folders
ii. Use ‘Stack to Images’ function to separate each image into individual colour channels
iii. Adjust Brightness/Contrast to a set level across all images
iv. Save the individual channels with correct Brightness/Contrast settings
v. Select ‘Color- Merge channels’ to re-create a multichannel image
vi. Change image type to ‘RGB color’
vii. Save this Merge image for each timepoint
viii. Once all merge images have been created/saved, Import Image Sequence selecting the first time point to create a stack of all timepoints
*When tiled images have been collected and stitched for each timepoint, this automatic function will not work due to the slight difference in overall image size due to the stitching process. Instead, open each of the separate merge images and then apply ‘Images to Stack’, using ‘Copy Centre’ function*
ix. Add Scale Bar – analyse – tools-scale bar
x. Add timestamp- image- stacks-timestamper
p. Save as AVI – uncompressed- 2fps

## 4 Results

### 4.1 Overview of the ALFA-PRF technique

**Figure 1:**
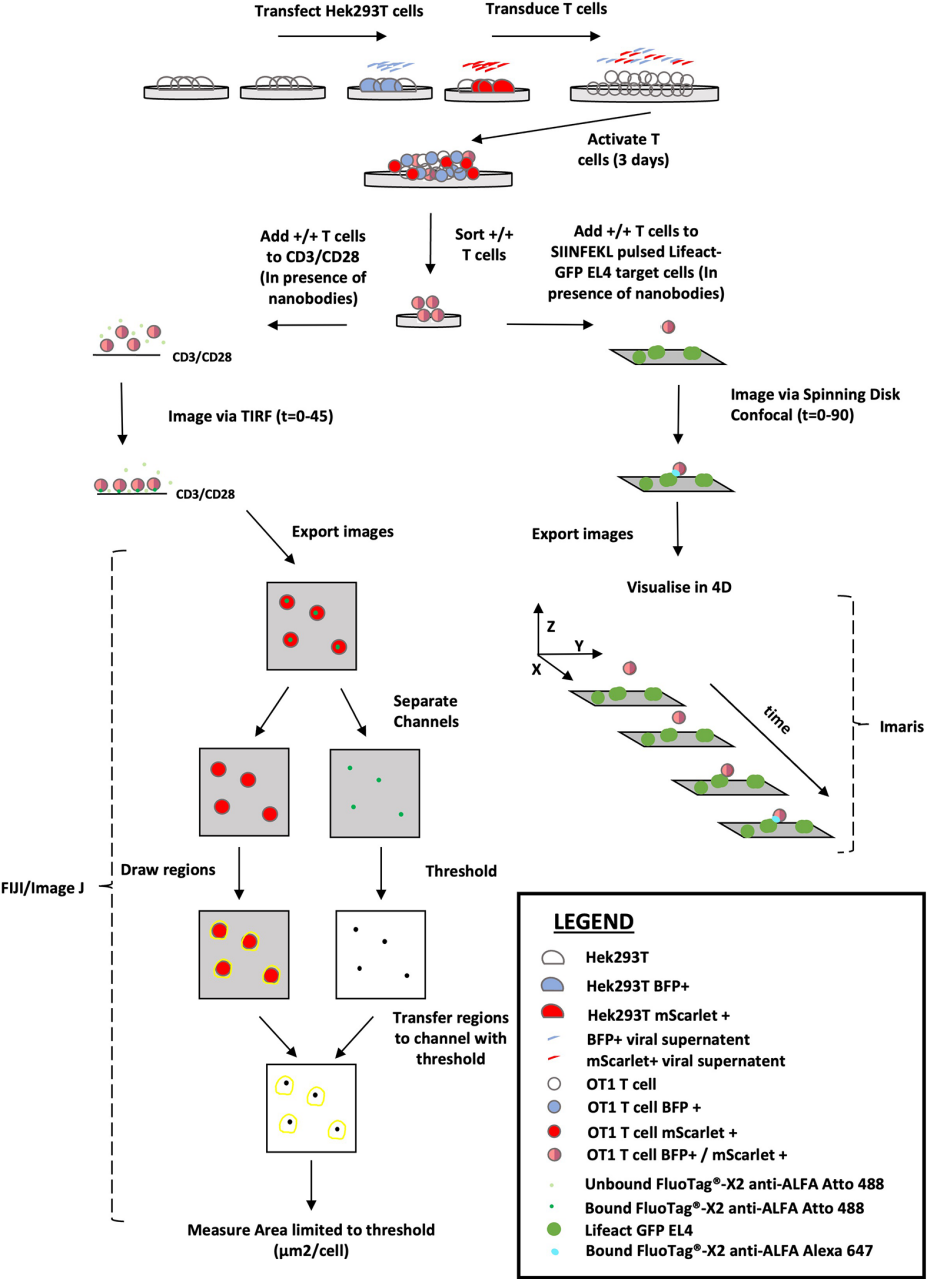
Overview of the ALFA-PRF Technique. Virus produced from HEK293T cells transfected with either MSCV-ALFA-PRF-TagBFP (BFP +) or MSCV-Lifeact-mScarlet (mScarlet +) is pooled and used to transduce OT1 T cells on Day 0 of activation. After 3 days of activation, the transduced T cells are sorted *via* flow cytometry to collect a pure population of double positive (+/+) cells. These double positive cells are then utilised in one of two ways: 1) added to CD3/CD28 coated imaging chambers in the presence of FluoTag^®^-X2 anti-ALFA Atto 488 nanobody and imaged *via* TIRF for 45 minutes. Resultant images are exported as OME TIFFS and opened in FIJI/Image J. Channels are separated so regions can be drawn around the lifeact-mScarlet signal, and then these regions are transferred to the thresholded (to highest grey value within synapse area in the Empty Vector control) FluoTag^®^-X2 anti-ALFA Atto 488 Channel image. An area measurement limited to threshold is then performed to obtain a value of perforin signal (µm^2^/cell). 2.) Added to SIINFEKL pulsed EL4 target cells that are also transduced with Lifeact-GFP, in the presence of FluoTag^®^-X2 anti-ALFA Alexa 647 nanobody and the immune synapse interaction visualised by spinning disk confocal microscopy to generate 4D data.

We have developed a novel technique to visualise perforin release during degranulation of murine CD8 T cells. Thus, a nanobody recognition tag cloned onto recombinant perforin (retrovirally transduced into the cells alongside a fluorescent actin or tubulin reporter) was detected by highly specific fluorescent nanobodies present in solution during synapse formation. The versatility of this approach is demonstrated by ALFA-PRF detection using both TIRF imaging of T cells forming synapses with a stimulatory anti-CD3/CD28 coated surface, and spinning disk confocal imaging of T cells forming synapses with antigen-presenting target cells. The perforin signal obtained using TIRF can then be quantified as an area measurement/cell using FIJI (27)/ImageJ software, and the perforin signal obtained using spinning disk confocal microscope can be visualised in 3D using Imaris software. The workflow of the technique is summarised in **Figure 1**, and a comprehensive validation of the system is detailed herein.

### 4.2 Cloning strategy

To avoid tagging perforin at the cleavable carboxy-terminus (21), we considered incorporating a tag into the amino-terminal region of perforin, where the tag would remain fused to perforin regardless of the action of proteases present in the secretory granules. Based on the crystal structure of perforin (28), we considered that a small amino-terminal tag was unlikely to interfere with perforin membrane binding or oligomerisation.

**Figure 2:**
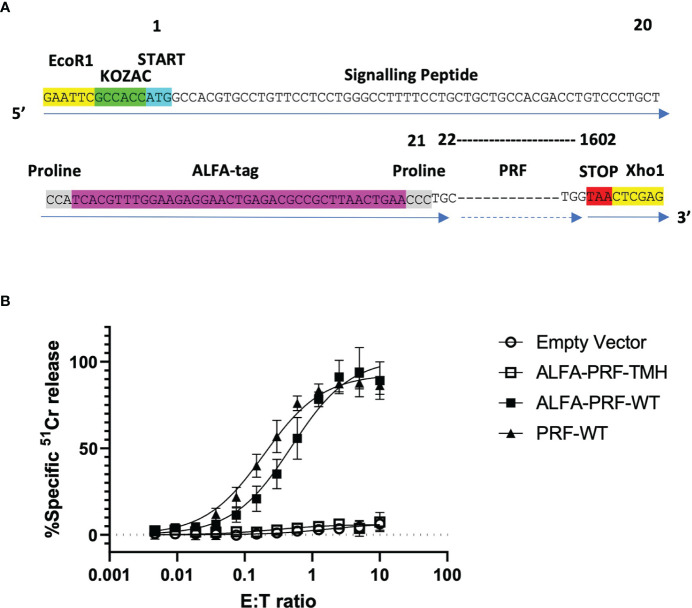
WT ALFA-PRF retains cytolytic function. **(A)** ALFA-tag sequence flanked by prolines is designed to reside immediately following the cleavable signalling peptide of perforin is cloned into MSCV-TagBFP using the restriction enzyme cut sites EcoR1 and Xho1. **(B)** Transduction of non-cytotoxic *Prf1^-/-^*.OT1 T cells with PRF-WT and ALFA-PRF-WT restores their killing capacity, while ALFA-PRF-TMH (‘disulphide locked’ perforin mutant) and Empty Vector do not restore function. N=3-6, error bars represent s.e.m. Individual values are shown in **Supplementary Table 1**.

Instead of engineering a bulky fluorophore into the N-terminal position (which would almost inevitably have affected the properties and stability of perforin), or a short linear peptide, which could be degraded by the lysosomal protease(s), the small ALFA-tag recognition sequence (SRLEEELRRRLTE) (23) was chosen as an appropriate alternative. This compact (α-helical) sequence can be detected by fluorescently-labelled, highly specific, small and, potentially, easily diffusible into the synapse FluoTag^®^-X2 anti-ALFA nanobodies (23). Due to the presence of a signalling peptide at the amino-terminus of perforin (amino acid positions 1 to 20), we engineered the tag immediately after that cleavable region, at amino acid position 21. To minimise the chance of any potential interference from neighbouring secondary structures of perforin, an N terminal ALFA-tag was surrounded by proline residues at the amino- and carboxy- termini of the peptide (23), resulting in the sequence shown in **Figure 2A**. Once the ALFA-tag had been engineered into the wild-type (WT) murine perforin sequence, we then cloned this construct into the EcoR1/Xho1 sites of the MSCV-IRES-TagBFP retroviral vector, resulting in the final DNA construct of MSCV-ALFA-PRF-WT-TagBFP. In addition to the wild-type construct, an amino-terminal ALFA-tag TMH ‘disulphide locked’ Prf mutant (26) was included as a non-lytic negative control (MSCV-ALFA-PRF-TMH-TagBFP), and an untagged wild-type (WT) Prf (MSCV-PRF-WT-TagBFP) was included as a positive control.

*Prf1^-/-^*.OT1 T cells were transduced with these constructs, sorted three days later to achieve equal TagBFP reporter expression level, and their ability to restore T cell cytotoxicity was assessed using a ^51^Cr release assay with syngeneic SIINFEKL-labelled EL4 targets. Whilst both the empty vector and the ALFA-PRF-TMH mutant had no cytotoxic activity, the ALFA-PRF-WT and the untagged PRF-WT control both restored function to similar levels (**Figure 2B**). This confirmed that the amino-terminal ALFA-tag did not affect the ability of perforin to form pores.

### 4.3 ALFA-tagged perforin is only detected upon its release from the cell

**Figure 3:**
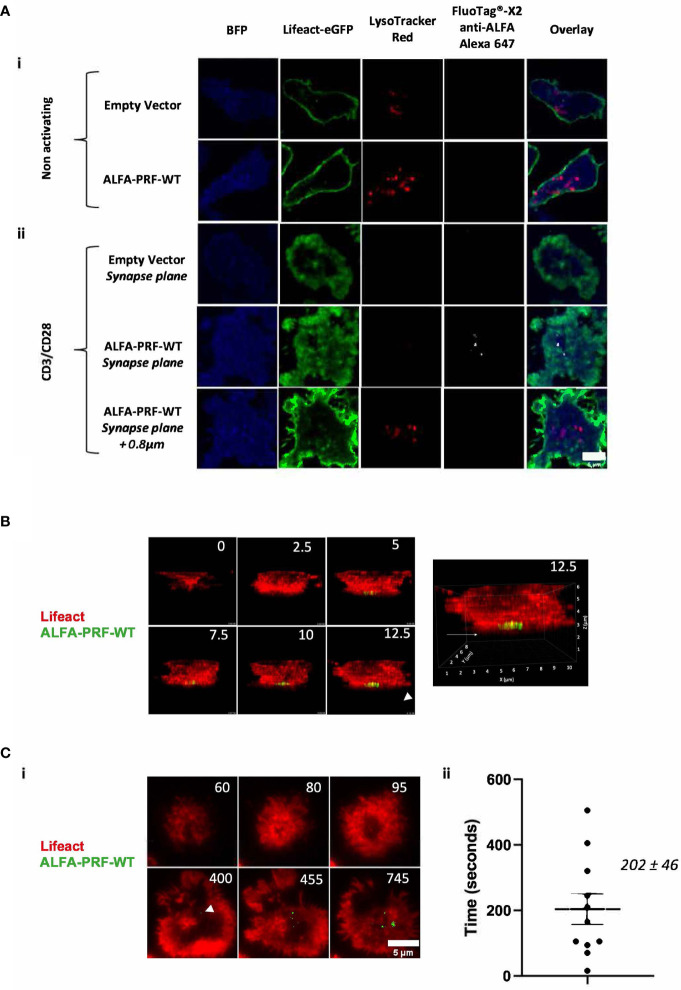
ALFA-PRF-WT detection by fluorescent nanobodies corresponds to its release from the cell **(A)** i.) ALFA-PRF-WT and Empty vector (EV) expressing T cells were imaged *via* confocal microscopy at the plane of the nucleus on a non-stimulatory surface. No ALFA-PRF-WT signal (detected by FluoTag^®^-X2 anti-ALFA Alexa 647, shown in white) is present in the lysosomal compartments (labelled with LysoTracker Red DND-99). **(A)** ii.) 45 minutes after transferring the cells from (i.) onto a stimulatory CD3/CD28 surface and imaging *via* confocal microscopy using a 2µm stack with 200nm intervals centred on the synapse plane, ALFA-PRF-WT signal was only observed at the plane of the synapse formation, and not 0.8µm above; conversely, no lysotracker signal was detected at the plane of the synapse. No FluoTag^®^-X2 anti-ALFA Alexa 647 signal is observed in the Empty Vector control. Cells shown are representative images from each field of view. Scale bar = 5µm. Empty vector control - n=3 biological replicates (mice), ALFA-PRF-WT n=2 biological replicates. **(B)** 3D confocal microscopy timelapse montage of Lifeact-mScarlet/ALFA-PRF-WT positive T cells synapsing upon a CD3/CD28 coated glass coverslip surface. ALFA-PRF-WT signal (shown in green, detected by FluoTag^®^-X2 anti-ALFA Atto 488) is detected only at the plane of the coverslip. Time is shown in minutes and enlarged view of time 12.5 (right) is shown to display orientation markers. Image reproduced from (24). **(C)** i.) Lifeact mScarlet/ALFA-PRF-WT positive cells were imaged in presence of FluoTag^®^ X2 anti-ALFA Atto 488 nanobody with imaging intervals of 5 seconds/frame. White arrow head indicates point of first ALFA-PRF-WT detection **ii.)** Time between initial actin clearance and Prf detection was measured for 11 cells, with a mean value of 202 ± 46 seconds (s.e.m., n=11). Scale bar = 5µm. For analysis, FluoTag^®^ X2 anti-ALFA Atto 488 signal was thresholded to the maximum value within an Empty Vector control. Individual values are shown in **Supplementary Table 2**.

If the nanobody was endocytosed and able to reach the ALFA-PRF within the secretory vesicles before the T cell formed a synapse, distinguishing the intracellular and extracellular fluorescence of ALFA-PRF during degranulation might be extremely challenging. To test for this, cells expressing Lifeact-eGFP and ALFA-PRF-WT were labelled with LysoTracker-Red DND-99 (to detect vesicular compartment) and imaged *via* confocal microscopy in the presence of FluoTag^®^-X2 anti-ALFA Alexa 647 nanobodies but in the absence of the anti-CD3/28 stimulus (**Figure 3A**.i.). Images were acquired in the middle plane of the cells, where LysoTracker positive granules could be seen. No FluoTag^®^-X2 anti-ALFA Alexa-647 fluorescence was detected within the lysotracker-positive compartments of either EV or ALFA-PRF-WT expressing cells, or inside the cells at all.

These exact same cell groups were then transferred from the non-stimulatory microscopy chamber to a glass bottom chamber slide containing immobilised anti-CD3/CD28 antibodies, and imaged 45 minutes later across a range of approximately 2µm, centred on the plane of the coverslip (**Figure 3A**.ii.). Whilst no FluoTag^®^-X2 anti-ALFA Alexa-647 fluorescence was observed in the empty vector group, punctate regions of bright FluoTag^®^-X2 anti-ALFA Alexa-647 fluorescence were observed in the ALFA-PRF-WT expressing cells. These punctate regions of fluorescence were located in the bottom plane of the cell within regions of actin depletion, which have previously been shown to be the location of granule secretion (29). This suggested that ALFA-PRF-WT release was only detectable upon synapse formation and degranulation. Similar results were obtained with the non-lytic ALFA-PRF-TMH (**Supplementary Figure 3**). Interestingly, lysotracker did not colocalise with ALFA-PRF in the synapse plane. The inability to detect lysotracker in that plane could be due to the nature of the confocal imaging, as opposed to previous reports visualising lysotracker at the immune synapse using TIRF (4), and/or due the fact that ALFA-PRF was only detected after the release from the secretory vesicles into the synaptic cleft. Additionally, these images were taken 45 minutes after addition of the cells to the CD3/CD28 coated surface, when T cells may have already released their secretory vesicles, and/or endocytosed them away from the immune synapse.

To ensure the FluoTag^®^-X2 anti-ALFA nanobodies were indeed detecting only secreted ALFA-PRF, and not ALFA-PRF contained within granules inside the cell which have docked at the membrane, Lifeact-mScarlet/ALFA-PRF-WT expressing T cells were imaged *via* 3D confocal microscopy in the presence of FluoTag^®^-X2 anti-ALFA Atto 488 nanobodies whilst they formed synapses on a stimulatory CD3/CD28 coated glass surface. It was found that FluoTag^®^-X2 anti-ALFA Atto 488 signal appeared at the bottom plane of the cell (**Figure 3B**), rather than throughout the cell as would be expected if the nanobodies were bound to the ALFA-PRF within the granules which relocate towards the synapse.

As an additional control for internalisation of the nanobody, Lifeact-mScarlet/ALFA-PRF-WT expressing T cells were incubated for 1 hour with FluoTag^®^-X2 anti-ALFA Atto 488 nanobodies without exposure to CD3/CD28 (to allow for the same amount of internalisation as would occur during the standard imaging). Following extensive washing to remove any extracellular nanobody, these cells were then imaged *via* TIRF upon addition to CD3/CD28. Unlike the cells imaged in the presence of the nanobody, where FluoTag^®^-X2 anti-ALFA Atto 488 signal appeared and increased over time (**Supplementary Figure 4i**, **Supplementary Movie 1**), no FluoTag^®^-X2 anti-ALFA Atto 488 was observed upon synapse formation in the washed cells (**Supplementary Figure 4ii**, **Supplementary Movie 2**). It was thus confirmed that ALFA-PRF detection during synapse formation was in fact due to the release of ALFA-PRF from the cells.

Having established this, we then increased the temporal resolution of our imaging to allow measurement of the time between actin clearance from the synapse and perforin release. We found that the average time between actin clearance and the first perforin detection was 202 ± 46 seconds (s.e.m., n=11) (**Figure 3C**, **Supplementary Movie 3**).

### 4.4 Quantification of ALFA-PRF release into the immune synapse

**Figure 4:**
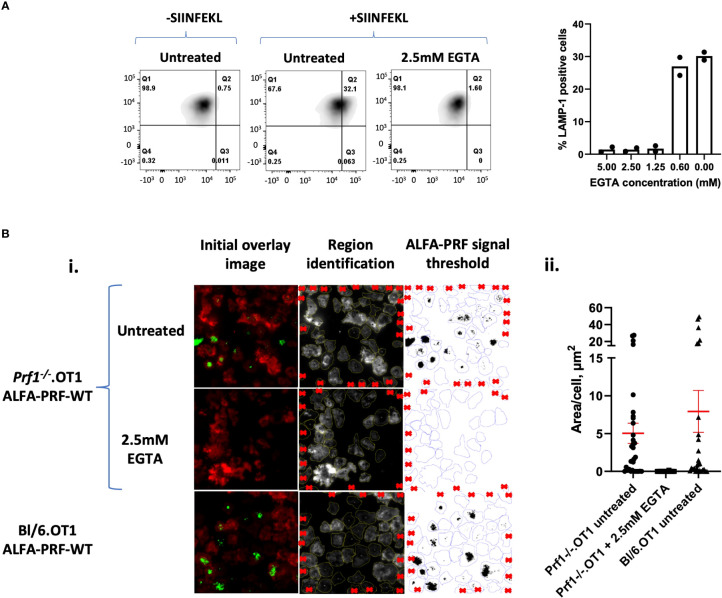
Quantification of ALFA-PRF-WT released into the synapse. **(A)** Degranulation assay (detecting LAMP-1 externalisation) shows inhibition of degranulation at 2.5 mM EGTA. Individual values are shown in **Supplementary Table 3**. N=2 biological replicates (mice). **(B)** (i) *First column:* TIRF microscopy images of *Prf1^-/-^*.OT1 cells expressing Lifeact-mScarlet (Red) and ALFA-PRF-WT (Top and middle rows) or Bl/6.OT1 expressing Lifeact-mScarlet (Red) and ALFA-PRF-WT (bottom row) after 45 minutes incubation on an anti-CD3/CD28 coated surface to induce synapse formation. ALFA-PRF-WT expressing *Prf1^-/-^*.OT1 cells were untreated or treated with EGTA. ALFA-PRF-WT is detected by FluoTag^®^-X2 anti-ALFA Atto 488 nanobodies (green). *Second column:* Lifeact-mScarlet and ALFA-PRF-WT channels were separated, regions drawn around the cells (Lifeact-mScarlet signal) and cells on edge regions noted for exclusion (red crosses). *Third column:* Regions were then transferred to the ALFA-PRF-WT channel and the signal thresholded to the maximum grey value within cell regions on the ALFA-PRF-WT channel of the Empty Vector control image (Empty Vector control image is shown in **Supplementary figure 5**). ii.) Thresholded images presented in i.) were analysed ‘limited to threshold’ to give values of perforin area per cell region. Mean values ( ± s.e.m. of n cells from one representative experiment) are: *Prf1^-/-^*.OT1 untreated - 5.045 ± 1.332 (n=32); *Prf1^-/-^*.OT1 +2.5mM EGTA - 0.006 ± 0.006 (n=27); Bl/6.OT1 untreated - 7.933 ± 2.772 (n=27). “n” refers to individual cells within the field of view, where regions were identified/excluded as per **Figure 5B**.i. Individual values are shown in **Supplementary Table 4**, from one mouse.

To demonstrate further applications of the nanobody-based system, we then proceeded to assess ALFA-PRF release from T cells at various conditions. For this, we chose to perform measurements at a single timepoint of 45 minutes, to allow for clear perforin detection across cells which had synapsed at various times during the incubation. To first establish a negative control for these microscopy experiments, we tested an inhibitor of T cell degranulation, a calcium chelator ethylene glycol-bis(β-aminoethyl ether)-N,N,N′,N′-tetraacetic acid (EGTA) by flow cytometry, which, as expected, efficiently inhibited exocytosis of the secretory vesicle (**Figure 4A**) without affecting cell viability. In the subsequent TIRF experiments, it was found that while untreated Lifeact-mScarlet/ALFA-PRF-WT-TagBFP double-positive cells were seen to release ALFA-PRF-WT (**Figure 4B**.i top row, **Supplementary Movie 4**), no ALFA-PRF-WT secretion was observed in the presence of 2.5mM EGTA (**Figure 4B**.i middle row, **Supplementary Movie 5**). Importantly, in agreement with previous studies using murine T cells (30), EGTA treated cells still formed a synapse, as confirmed by clearance of Lifeact-mScarlet signal indicating actin depletion (**Supplementary Figure 5**). Cells expressing empty vector instead of ALFA-PRF-WT showed actin clearance without nanobody signal (**Supplementary Figure 6**, **Supplementary Movie 6**). As *Prf1^-/-^*.OT1 mice may not be available as readily as Bl/6.OT1 mice, we explored ALFA-PRF secretion in perforin-competent T cells, and found that the endogenous protein does not interfere with its release and detection in the immune synapse (**Figure 4B**.i. bottom row; **Supplementary Movie 7**). These images were analysed and quantified as described in Methods Section 6 (**Figure 4B**.ii).

**Figure 5:**
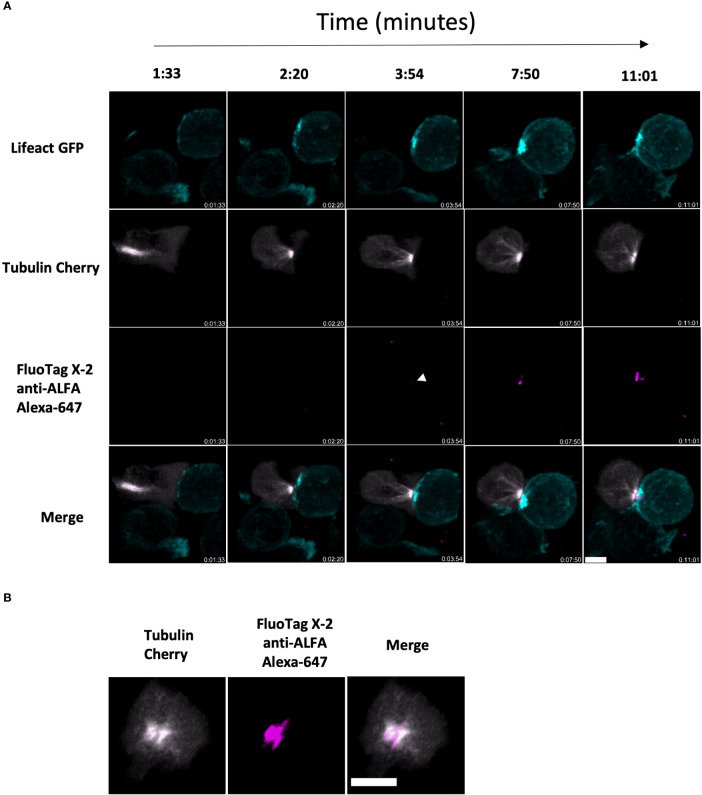
ALFA-PRF-TMH release can be detected within a bona fide synapse between CD8 T cell and antigen presenting target cell **(A)** Side on montage showing release of ALFA-PRF-TMH (magenta) in the area between the polarised MTOC of mCherry-tubulin expressing *Prf1^-/-^*.OTI CD8 T cells (grey) and the Lifeact GFP expressing EL4 target cell (cyan). White arrow head indicates point of first ALFA-PRF-TMH detection. Synapse interaction shown is a representative example from three fields of view (containing >5 synapse interactions each) of two biological replicates (mice). **(B)** En face view of the synapse from timepoint 11:01 in a.) showing the location of the Perforin within the synapse area. For clarity, brightness and contrast of images has been altered. Scale bars = 5µm.

Next, we explored the release of ALFA-PRF in the context of the physiological immune synapse, in a similar manner to how Syb2-mRFP has been detected by anti-RFP antibodies previously (31). To test this, we employed *Prf1^-/-^*.OT1 CD8 T cells, which have been shown to form stable synapses due to their inability to kill the target cells (32) thus expanding the window of opportunity for imaging the synapse. The cells were co-transduced with Tubulin-mCherry and the non-lytic ALFA-PRF-TMH. Antigen-presenting (SIINFEKL) EL4 cells expressing Lifeact-GFP were immobilised on a glass cover-slip by briefly incubating them in the serum-free media. Effector cells were then added in a complete media, and images were collected over time using Spinning Disk confocal microscope. We detected synapse formation between the two cell types by the docking of the MTOC (Cherry-tubulin) at the point of contact with the target cells, and this was followed by the release of ALFA-PRF-TMH into the synapse (as detected by anti-ALFA Alexa-647 nanobodies) (**Figure 5** and **Supplementary Movie 8**).

**Figure 6:**
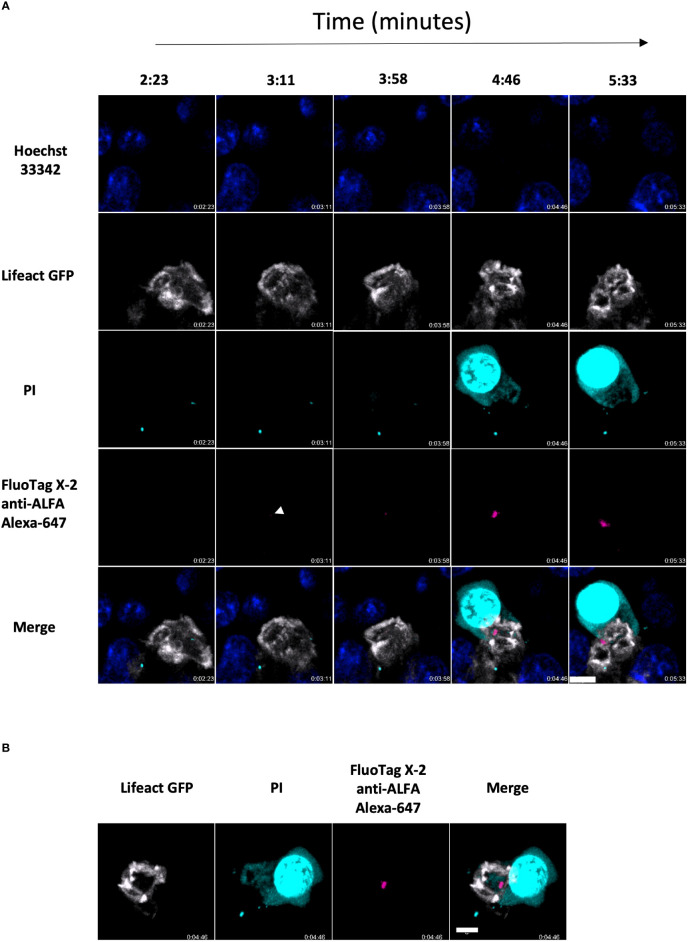
Synchronisation of ALFA-PRF-WT release from CD8 T cell with permeabilisation of the target cell membrane. **(A)** Side on montage showing release of ALFA-PRF-WT (magneta) in the area within the ring of actin depletion in the Lifeact GFP expressing Prf ko OTI CD8 T cells (grey) and the Hoechst 33342 labelled EL4 target cell, just prior to PI blush (cyan). White arrow head indicates point of first ALFA-PRF-WT detection. Shown is a representative example from two fields of view (containing >5 synapse interactions each) of one biological replicate (mouse). **(B)** En face view of the synapse from timepoint 4:46 in a.) showing the location of the Perforin within the synapse area. Note, Hoechst 33342 was imaged only in the middle plane of the image, therefore was removed from display in this en face projection. Scale bars = 5µm.

Finally, having previously observed perforin-mediated disruption of the target cell membrane (using media supplemented with 100µM propidium iodide; Lopez, Blood 2013), but without visualising perforin itself (due to the lack of an appropriate methodology), we now wished to test whether ALFA-PRF and membrane disruption can be detected concurrently. To do this, we used *Prf1^-/-^*.OT1 CD8 T cells co-expressing Lifeact-GFP and ALFA-PRF-WT, and imaged their interaction with SIINFEKL antigen-pulsed and Hoechst-labelled EL4 cells in the media supplemented with 100µM Propidium Iodide and anti-ALFA FluoTag-X2 Alexa 647 nanobodies. Indeed, we were able to visualise ALFA-PRF-WT release just prior to propidium iodide influx into the target cells (**Figure 6** and **Supplementary Movie 9**), thus providing a direct evidence that the target cell membrane disruption is dependent on the secreted perforin.

## 5 Discussion

Increased understanding of T cell biology has led to many advances in medical research, with one of the most prominent examples being Chimeric Antigen Receptor (CAR) T cell therapy to combat various blood cancers (33, 34). However, the limited success of CAR-T cell therapy in other settings, such as treatment of solid tumours (35) highlights the fact that much still remains to be learnt about T cells fundamental biology. Many of these remaining unknowns involve the two key effector molecules of CD8 T cells, perforin and granzyme B. To understand these molecules better, model murine systems are often employed, where specific genes of interest can be knocked out and their effect on T cell function studied. Unfortunately, detection of murine perforin has remained a challenging issue for T cell biology researchers, due to both its low abundance in murine T cells, and the lack of a sufficiently sensitive antibody. To overcome this problem, we have developed a novel version of murine perforin incorporating an ‘ALFA-tag’ nanobody recognition sequence to the N terminal of the protein. This unique system avoids the otherwise problematic issue of perforin carboxy-terminal proteolysis (21), and in combination with a fast and sensitive imaging approach based on TIRF microscopy or other imaging techniques, such as spinning disk microscopy used here, also allows for the use of highly specific FluoTag^®^-X2 anti-ALFA nanobodies.

The use of nanobodies instead of traditional antibodies solves a potentially significant problem of size exclusion within the immunological synapse (36): since nanobodies (4x2.5nm) are considerably smaller than standard antibodies (10nm), they are expected to diffuse more readily into the synaptic cleft. With that said, anti-RFP antibodies (31) and fluorescently labelled Annexin (3) have both been shown previously to label proteins/lipids within the synapse area in live cell imaging. Additionally, we have recently used anti-GzmB antibodies to detect GzmB release within the synapse (24). While the detection of endogenous granzyme B may be attributed to its very high expression level and/or high affinity of antibodies, the use of a nanobody detection system may be more suitable for low abundance proteins such as murine perforin explored here, or similarly where no reliable antibody exists for the protein of interest.

We have hereby demonstrated the capability of the ALFA-PRF system to quantitatively assess perforin release from live T cells that have formed synapses on anti-CD3/CD28 coated glass coverslips. Using a series of comprehensive control experiments, we have also demonstrated that ALFA-PRF (both WT and TMH) can only be detected once it is released into the synapse. We show that the average time between actin clearance and ALFA-PRF-WT release was 202 seconds (**Figure 3C**ii). This was remarkably consistent with a previous study exploring the release of the recombinant GzmB-TFP fusion protein from murine T cells on lipid bilayers (37). In this study, the time between initial CTL adhesion and vesicle secretion was found to be 285 seconds. However, the delay between CTL contact and actin clearance was estimated to be 106 seconds, so a timing of 179 seconds between actin clearance and perforin delivery was very close to the value of 202 seconds reported in the current study. This is also consistent with the previously measured time interval between calcium flux into the effector cell and perforin-mediated disruption of the target cell membrane (2). Importantly, the current experimental system is able to detect the release of ALFA-PRF not only from *Prf1^-/-^*.OT1, but also from the more readily available wild-type BL/6.OT1 mice. In conjunction with the simple workflow to produce the ALFA-PRF expressing cells, this makes our system highly accessible (and versatile) to many researchers in the field. Similarly, as the image analysis pipeline utilises the freely available image processing software FIJI/ImageJ, no expensive subscription analysis software is required to process the images.

Endogenous perforin expression level is relatively low in T cells, and this (as well as the lack of appropriate antibodies) may be the main reasons for not being able to detect perforin release into the immunological synapse. Therefore, in order to detect ALFA-PRF, we overexpressed it under a strong CMV promoter, and it is therefore likely that the levels of ALFA-PRF observed in this study are higher than endogenous protein. It is therefore possible that detection of ALFA-PRF may be hindered/reduced if ALFA-PRF were expressed at endogenous levels using a knock-in Mouse model. This is in contrast with a high endogenous level of of GzmB, and as a result its secretion can be readily detected by primary antibodies (24) or by using a knock-in mouse model (38). Nevertheless, by inhibiting CTL degranulation, we demonstrate that the current overexpression system is not “leaky”, and offers a reliable approach to assessing perforin secretion into the immunological synapse in real time.

With the ability to detect murine perforin secretion, we believe this paves the way to investigate novel aspects of secretory vesicle exocytosis. Although a detailed pursual of all possible measurements are outside the scope of this study, as a proof of principle we have shown the detection of ALFA-PRF released from CD8 T cells is not limited to an artificial synapse, and it can also be detected within a physiological synapse. Whilst we here provide the evidence using spinning disk confocal microscope, other experimental setups can be used for exploring *bona fide* synapse interactions. We anticipate that many further discoveries may now be possible using, for example, highly advanced four dimensional imaging systems such as that provided by Lattice Light Sheet microscopy (39), where it has already been demonstrated that T cells can be imaged during conjugation with target cells (29).

Overall, we present a novel methodology for direct, quantifiable measurement of perforin released from live murine T cells during synapse formation. This advancement should be useful in deciphering the behaviour of CTLs from various mouse models of disease, allowing differentiation of diseases where cytotoxic machinery is impaired and perforin not released (40, 41), to those where perforin is released but inactivated (24). More generally, the use of nanobodies for detecting low abundance protein secretion into the immunological synapse may be a viable strategy for an otherwise very challenging field of research.

## Data availability statement

The original contributions presented in the study are included in the article/**Supplementary Material**. Further inquiries can be directed to the corresponding authors.

## Ethics statement

The animal study was reviewed and approved by Peter MacCallum Cancer Centre Animal Experimentation Ethics Committee (AEEC) E655.

## Author contributions

JARS and IV conceived the study, JARS designed and performed the experiments, RFL assisted in developing and optimising the Live Cell TIRF technique and contributed to writing the manuscript. TN performed the experiments and contributed to writing the manuscript, AJB assisted in developing and optimising the image analysis pipeline and contributed to writing the manuscript, JARS and IV co-wrote the manuscript, IV supervised the study.
