# Supplementary material for: ALFA-PRF: a novel approach to detect murine perforin release from CTLs into the immune synapse
Source: Front Immunol. 2022 Dec 22;13:931820. doi: 10.3389/fimmu.2022.931820 (PMC9813862; doi:10.3389/fimmu.2022.931820)
Supplement: Supplementary file 10 [file DataSheet_1.pdf]

Supplementary Information

Supplementary Methods

MSCV-Lifeact-eGFP cloning (Figure 3a)

- Lifeact-eGFP fusion possessing 5' EcoR1 and 3' Sal1 restriction enzyme cut sites (Integrated DNA Technologies) was cloned into MSCV-IRES-GFP construct between EcoR1 and Sal1 (hence removing the fluorescent reporter of the vector)

^{51}Cr release assay (Figure 2b)

- This figure is reproduced from (Castiblanco et al., 2022). Chromium Assay description is provided in the initial publication.

LysoTracker™ labelling/microscopy (Figure 3a)

- Cells were labelled with 75nM LysoTracker™ Red DND-99 (Thermo Fisher Scientific, Massachusetts, USA) for 1.5 hours at 37 degrees, before being pelleted and resuspended in fresh DMEM media containing 50nM Fluo-tag X2 anti-ALFA Alexa 647 (NanoTag Biotechnologies GmbH, Göttingen, Germany).

3D Confocal Microscopy (Figure 3b)

- This image is reproduced from (Castiblanco et al., 2022). Briefly, T cells expressing Lifeact-mScarlet/ALFA-PRF-WT were added to CD3/CD28 coated coverslips and imaged via 3D confocal microscopy during synapse formation in the presence of (FluoTag®-X2 anti-ALFA Atto 488).

Degranulation Assay (Figure 4a)

- 1M EGTA stock solution was diluted in H_20 to 20x stocks of desired concentrations.
- 10µl of these concentrate (20X) stocks added into 96 well plate.
- T cells and EL4 labelled ± SIINFEKL were mixed at 1:1 volume ratio (4:1 cell number ratio, 200,000 EL4/50,000 T cells) to make a volume that equates to 200µl per condition.
- 200µl of this T cell/target mix was added to each well (on top of the 10µl H_20/EGTA).
- 4µl of PE anti-mouse CD107a (LAMP-1) antibody (Biolegend, San Diego, CA) was added to the cells and mixed by pipetting up and down.
- These samples were then incubate at 37 °C for 2 hours.
- After 2 hours, cells were pelleted and stained with anti-mouse CD8α APC for 20 minutes on ice.
- After washing, samples were analysed on FORTESSA X20 flow cytometery analysis machine.
- LAMP-1 positive cells within the CD8+ population were plotted using FlowJo analysis software.

Supplementary Figure 1:
Strong compensation guided by single colour controls is required to distinguish double positive Lifeact-mScarlet/BFP positive cells (highlighted by red circle in bottom row) via flow cytometry.

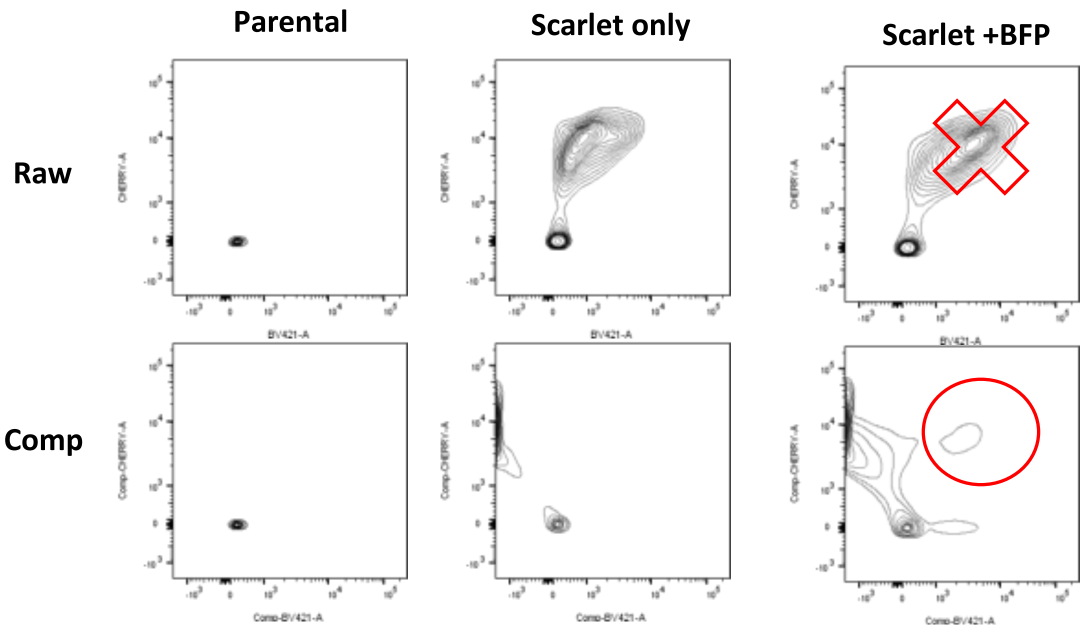

Supplementary Figure 2:
Focus on the plane of synapse formation can be set at early time-points by focusing on the probing microvilli (white arrows in T=0:00) of the T cells. Development of one individual synapse from scanning microvilli to classical actin ring can be followed by white asterisk across the 4 time frames.

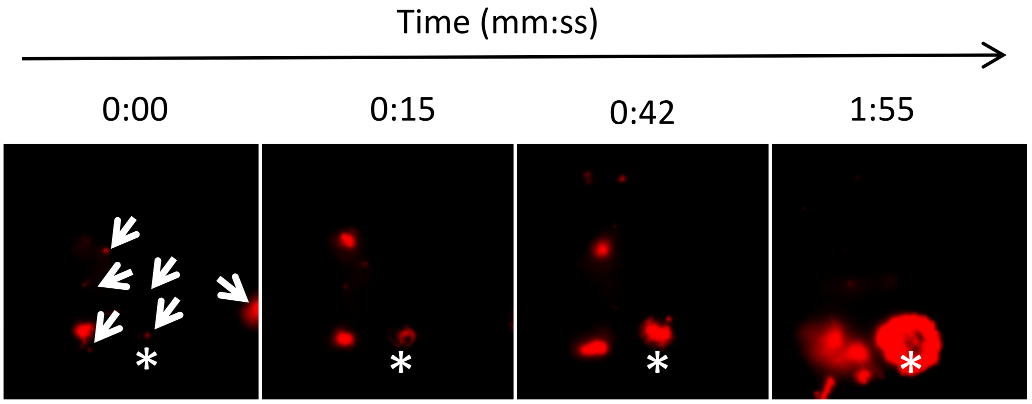

Supplementary Figure 3:
i). ALFA-PRF-TMH expressing T cells were imaged via confocal microscopy at the plane of the nucleus on a non-stimulatory surface. No ALFA-PRF-TMH signal (detected by FluoTag®-X2 anti-ALFA Alexa 647, shown in white) is present in the lysosomal compartments (labelled with LysoTracker Red DND-99).

ii) 45 minutes after transferring the cells from (i.) onto a stimulatory CD3/CD28 surface, ALFA-PRF-TMH signal is only observed at the plane of the synapse formation, not 0.8μm above. Cells shown are representative images from each field of view.

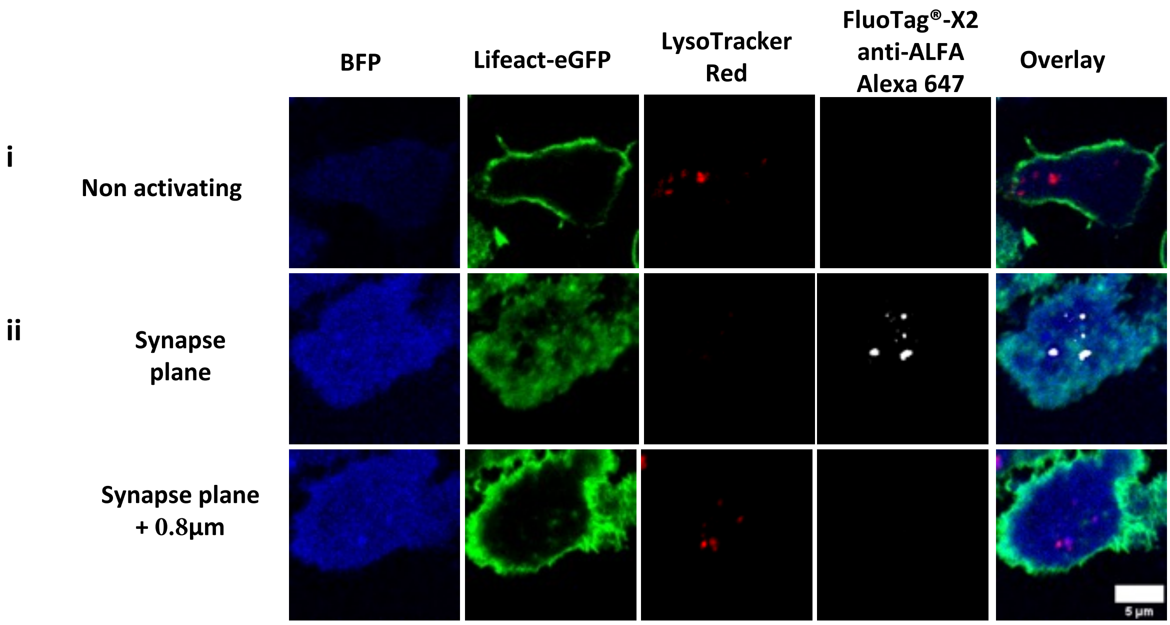

Supplementary Figure 4:

i.) Lifeact mScarlet (Red)/ALFA-PRF-WT positive cells were imaged in presence of FluoTag®-X2 anti-ALFA Atto 488 nanobody (green) for 1 hour at 37 degrees Celsius.

ii) A separate group of Lifeact mScarlet/ALFA-PRF-WT positive cells were incubated in the presence of FluoTag®-X2 anti-ALFA Atto 488 for the same period of time but then washed to remove all nanobody in solution before being added to CD3/CD28 coverslip and imaged by TIRF for an additional 45 minutes.

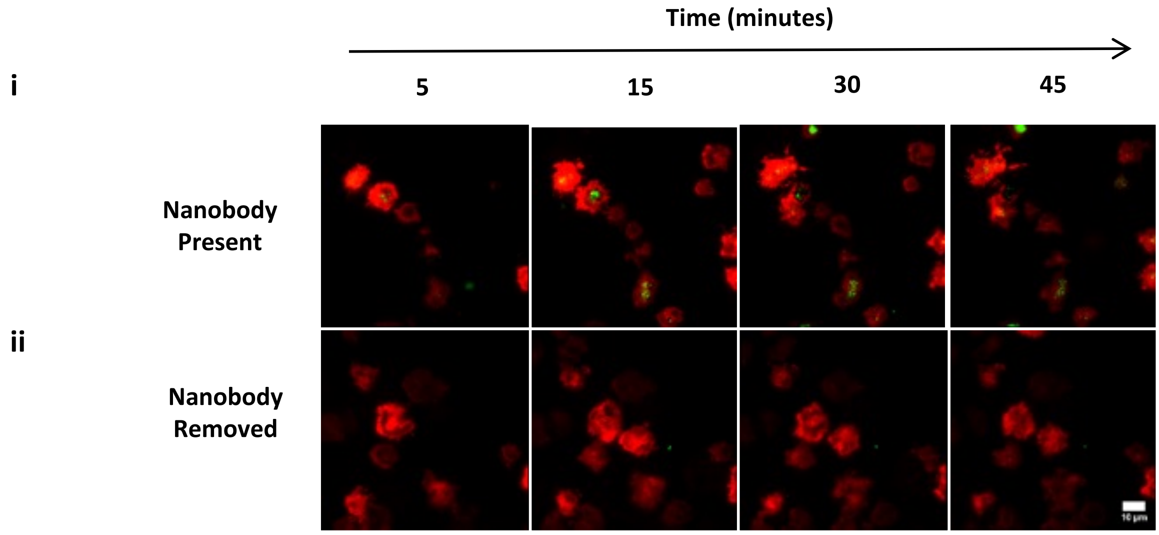

Supplementary Figure 5:
Regions of actin (Red, Lifeact-mScarlet) clearance (shown by white arrow heads) are seen throughout the *Prf* $^{-/-}$ OT1 ALFA-PRF-WT + 2.5mM EGTA time course microscopy experiment, indicating synapse formation. Representative timepoint shown is t = 20 minutes. Full time course is presented in *Supplementary Movie 4*.

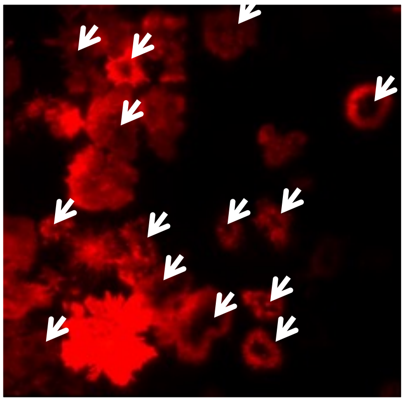

Supplementary Figure 6:
Cells expressing empty vector showed actin clearance (Red, Lifeact Scarlet) without nanobody signal (Green, FluoTag-X2 anti-ALFA Atto 488). Representative image from t=45 is shown, full time course is presented in *Supplementary Movie 6*.

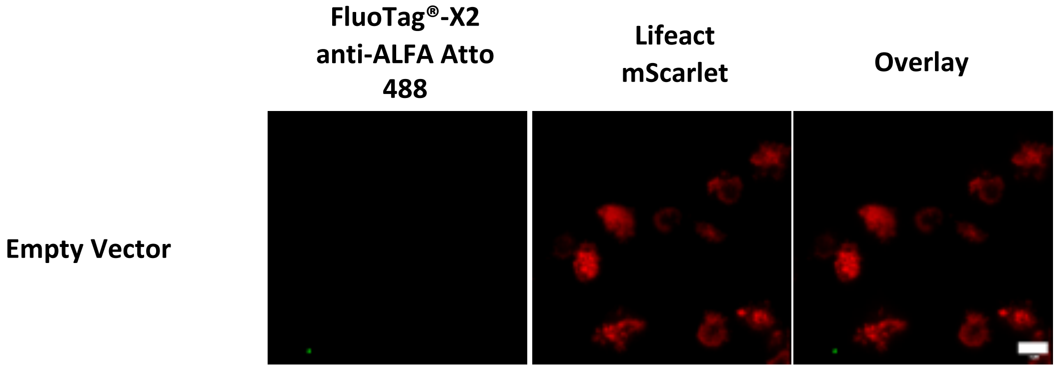

Supplementary Table 1. ^{51}Cr release assay (as shown in Figure 2b)

E/T ratio	Empty Vector						ALFA-PRF-TMH					ALFA-PRF-WT						PRF-WT		
**10**	4.99	1.07	8.25	11.32	3.51	8.42	1.93	5.83	7.86	16.50	4.68	80.52	109.95	80.86	85.25	87.74	90.37	80.55	90.34	87.82
**5**	4.24	0.02	4.58	8.85	0.87	7.61	3.07	-0.34	2.14	11.55	2.01	107.75	77.85	113.82	81.93	91.92	90.42	83.78	90.22	89.12
**2.5**	0.70	0.53	6.25	4.79	2.39	6.31	6.23	7.73	6.55	7.13	3.58	96.53	102.91	99.17	78.87	84.66	84.40	83.45	85.75	92.41
**1.25**	1.34	-0.74	6.49	3.29	2.52	5.10	5.89	5.86	6.84	4.21	0.93	81.06	80.51	82.76	70.73	77.59	77.08	79.22	84.06	86.79
**0.6**	0.68	-0.80	5.70	3.65	1.05	3.07	6.29	3.13	4.37	3.26	1.79	60.57	32.72	60.45	54.18	67.24	59.13	71.26	79.70	77.06
**0.3**	0.61	0.04	0.50	3.32	0.63	1.29	3.24	0.74	6.76	0.68	2.14	32.55	27.34	30.28	31.60	50.90	37.77	48.38	66.97	55.16
**0.15**	2.26	-0.38	-2.13	0.79	1.23	0.65	3.61	-0.50	4.93	-0.46	0.01	17.50	17.80	18.54	16.91	35.57	18.38	37.09	47.58	35.12
**0.075**	-0.49	-1.95	3.28	-0.39	-1.65	0.20	3.05	2.13	3.70	-0.55	0.43	9.50	8.90	10.55	7.88	19.25	11.96	19.66	28.13	17.56
**0.0375**	2.74	-3.25	5.20	-0.56	-1.27	-0.60	4.56	1.45	3.56	-0.19	-1.57	5.64	0.83	8.14	5.21	8.44	6.27	12.76	14.89	7.96
**0.01875**	0.09	-2.57	4.17				-0.15	0.95	4.07			3.41	4.97	5.43						
**0.00938**	1.34	-2.35	2.64				2.76	0.91	4.49			5.74	2.79	3.49						
**0.00469**	3.66	-2.71	0.93				0.32	2.26	4.37			2.44	0.80	4.45						

Supplementary Table 2. Time (seconds) between initial actin clearance and Prf release (as shown in Figure 3c.ii).

Prf1-/-.OT1 ALFA-PRF-WT
93.5
320
505
70
165
105
245
210
105
15
405

Supplementary Table 3. Results of LAMP-1 externalisation assay (as shown in Figure 4a).

	Rep1	Rep2
**5**	0.68	2.21
**2.5**	0.87	1.96
**1.25**	0.85	2.57
**0.6**	29.75	24.23
**0**	31.35	28.93

Supplementary Table 4. Quantification of ALFA-PRF-WT release (as shown in Figure 4b.ii).

Prf1-/-.OT1 untreated	Prf1-/-.OT1 + 2.5mM EGTA	Bl/6.OT1 untreated
0.57	0	7.15
6.37	0	0.45
1.89	0	17.65
0	0	0.12
0	0	4.22
0	0	0.42
0.14	0	21.63
27.46	0.01	0
4.8	0	0.31
3.74	0	0.42
20.9	0	0.84
7.28	0	0
3.3	0	1.47
16.4	0	0
10.12	0	0.06
0.02	0	0
0.01	0	0.96
7.82	0	0.28
3.64	0	0
0	0	36.29
1.33	0	1.04
1.24	0	4.86
0.16	0	49.3
7.04	0	0.19
4.13	0	17.29
26.63	0	46.51
3.13	0.15	2.72
1.4		
1.64		
0		
0.28		
0		

Supplementary Movies

Supplementary Movie 1: 45 minute timecourse (5 minute frames) corresponding to Figure 3c (top panel). An increase in Green fluorescence is observed over time, largely within the regions of actin depletion, when ALFA-PRF-WT/ Lifeact-mScarlet (Red) expressing T cells are imaged in the presence of FluoTag®-X2 anti-ALFA Atto 488 nanobodies.

Supplementary Movie 2:
45 minute timecourse (5 minute frames) corresponding to Figure 3c (bottom panel). No increase in Green fluorescence is observed over time when ALFA-PRF-WT/Lifeact-mScarlet (Red) expressing T cells, pre incubated with FluoTag®-X2 anti-ALFA Atto 488 nanobodies but washed thoroughly before addition to CD3/CD28 are imaged.

Supplementary Movie 3: 1125 second timecourse (5 second frames) corresponding to Figure 3c i. ALFA-PRF-WT/Lifeact-mScarlet (Red) expressing T cells imaged immediately following their addition added to CD3/CD28 in the presence of FluoTag®-X2 anti-ALFA Atto 488 nanobodies (Green).

Supplementary Movie 4: 45 minute timecourse (5 minute frames) corresponding to panel 1 of Figure 4b, demonstrating the detection of ALFA-PRF-WT with FluoTag®-X2 anti-ALFA Atto 488 nanobodies (Green), largely within regions of actin depletion, when ALFA-PRF-WT/Lifeact-mScarlet (Red) expressing *Prf1*$^{-/-}$.OT1 T cells form synapses on an activating CD3/CD28 surface.

Supplementary Movie 5: 45 minute timecourse (5 minute frames) corresponding to panel 2 of Figure 4b, demonstrating the absence of detection of ALFA-PRF-WT with FluoTag®-X2 anti-ALFA Atto 488 nanobodies (Green) when ALFA-PRF-WT/Lifeact-mScarlet (Red) expressing *Prf1*$^{-/-}$.OT1 T cells form synapses on an activating CD3/CD28 surface in the presence of 2.5mM EGTA.

Supplementary Movie 6: 45 minute timecourse (5 minute frames) corresponding to panel 3 of Figure 4b, demonstrating the absence of detection of ALFA-PRF-WT with FluoTag®-X2 anti-ALFA Atto 488 nanobodies (Green) when Empty Vector/Lifeact-mScarlet (Red) expressing *Prf1*$^{-/-}$.OT1 T cells form synapses on an activating CD3/CD28 surface.

Supplementary Movie 7: 45 minute timecourse (5 minute frames) corresponding to panel 3 of Figure 4ci, demonstrating the detection of ALFA-PRF-WT with FluoTag®-X2 anti-ALFA Atto 488 nanobodies (Green), largely within regions of actin depletion, when ALFA-PRF-WT/Lifeact-mScarlet (Red) expressing Bl/6.OT1 T cells form synapses on an activating CD3/CD28 surface.

Supplementary Movie 8: Time course demonstrating the detection of ALFA-PRF-TMH with FluoTag®-X2 anti-ALFA Alexa 647 nanobodies (magneta) within the synapse formed between a Cherry tubulin (grey) expressing *Prf1*$^{-/-}$.OT1 T cell and a Lifeact GFP expressing, SIINFEKL pulsed EL4 target cell (Cyan).

Supplementary Movie 9: Time course demonstrating the detection of ALFA-PRF-WT with FluoTag®-X2 anti-ALFA Alexa 647 nanobodies (magneta) within the synapse formed between a Lifeact Scarlet (grey) expressing *Prf1*$^{-/-}$.OT1 T cell and a Hoeschst labelled, SIINFEKL pulsed EL4 target cell (blue), just prior to the distinctive influx of Propidium Iodide (Cyan) upon membrane permeabilsation of the target cell.
